# Spatial mapping of soil properties in Konkan region of India experiencing anthropogenic onslaught

**DOI:** 10.1371/journal.pone.0247177

**Published:** 2021-02-19

**Authors:** Ram Ratan Verma, Tapendra Kumar Srivastava, Pushpa Singh, B. L. Manjunath, Anil Kumar

**Affiliations:** ICAR- Indian Institute of Sugarcane Research, Lucknow, India; Assam University, INDIA

## Abstract

Soils of Indian Konkan region, part of ecologically sensitive Western Ghats have been subjected to anthropogenic activities of late. This has endangered the ecological security through conspicuous losses in topsoil quality. The rationale of the present study was to map the soil properties and create management zones for ensuring food and nutritional security. The study was conducted in South Goa district of the state of Goa located in Konkan region. A total of 258 geo-referenced soil samples were collected and analyzed for pH, EC, SOC, available N, P, K and DTPA extractable micronutrients *viz*., Zn, Cu, Fe and Mn. Soil pH was found to be in acidic range. A wide variability existed in SOC content ranging from 0.12–5.85%. EC was mostly neutral with mean value 0.08±0.37 dSm^-1^, while available nitrogen (AN), available phosphorus (AP) and available potassium (AK) varied in range from 56.4–621.6 kg ha^-1^, 0.5–49.7 kg ha^-1^ and 31.5–786.2 kg ha^-1^ with mean values 211.2±76.9, 8.4±8.2 and 202.3±137.6 kg ha^-1^, respectively. A wide range was exhibited by cationic DTPA extractable Zn, Cu, Fe and Mn with mean values, 0.22±0.30, 0.44±0.60, 7.78±5.98 and 7.86±5.86 mg kg^-1^, respectively. Soil pH exhibited significant positive correlation with EC, AP AK and Zn and negative correlation with Fe and Cu. SOC exhibited significantly correlated with AN, AP, AK, Zn and Fe. Geo-statistical analysis revealed J-Bessel as best fit semivariogram model for pH, AP and AK; Rational Quadratic for EC, SOC, Zn and Mn; Hole effect for AN; Stable for Cu and K-Bessel for Fe for their spatial mapping. Four principal components showed eigenvalues more than one and cumulative variability of 59.38%. Three distinct soil management zones showing significant variation in soil properties were identified and delineated for wider scale management of soils. Precision nutrient management based on spatial variation and their mapping would enable refined agricultural and environmental management practices in the region.

## Introduction

Indian Konkan region is part of UNESCO recognized world heritage site ‘Western Ghats’, a global biodiversity hotspot, rich in biodiversity and species endemism [1, 2]. It is 50 km wide narrow coastal lowland with a sloping terrain, ecological charms, magnificent forest canopy and golden palm fringed beaches [3]. However, its environment is under tremendous pressure from human interference for developmental activities [4, 5]. Anthropogenic activities like mining, industrialization and infrastructure boom have dominated the region during past few decades. These have involved ruthless cutting of trees and caused removal of topsoil layer for creating sink holes for indiscriminate dumping of created rejects of mining in the soil [6]. Disturbance in structure of top soil layer has increased soil compaction, soil erosion and caused appreciable amount of adverse effects on soil physico-chemical and biological properties [7, 8]. Soil erosion at a large scale has led to deterioration of soil fertility, destruction of biodiversity and other societal resources [5, 9, 10]. Long term soil erosion completely exposes the top soil to air and releases large amount of carbon and nitrogen, which in turn changes the degraded ecosystems from C (N) sinks to C (N) sources [11]. Further, pouring of mine rejects containing toxic metals into agricultural soils and rivers have contaminated soil and water [5, 6]. Consequently, drastic disturbance in soil physico-chemical properties and nutrient dynamics have led to disastrous effects on nutritional and ecological security in the region. The present work was therefore taken up in South Goa district, a key location within the Konkan region, for addressing the declining soil quality *vis- a- vis* aggravating environmental stress.

South Goa is one of the two districts that comprise the state of Goa, where about 900 sq km of forests stand denuded and are now barren. The district covers an area of 1966 km^2^ with a forest cover stretching across 1302 km^2^ [3]. About 10 large mines are situated on the hillocks of South Goa that have created large pits in form of craters and exposed the unconsolidated soil surface. The mines generate about 3300 tonnes of rejected dumps every day on the hill slopes [5]. The rejects are mostly clay forms of soil containing toxic metals and are poured into the agricultural fields, Zuari river and other water courses located at low levels during the monsoons [4, 5]. It is reported that metals associated with mining possess toxic properties if present above certain levels [12]. Fe, Mn, Cu, Pb, Cd, Cr and Co are known as beneficial elements for plant growth, but all are phytotoxic at higher concentrations [13]. The deposition of toxic metals thus have altered soil physico-chemical properties and disrupted the surface and subsurface hydrologic regimes which in turn have affected the soil nutrients and sustainable ecological recovery adversely [14]. Further, alteration of soil properties influence the energy and substrates transformation and result in nutrient deficiency in soil [15]. The deposition and disturbances have modified the soil across the spatial and temporal scales [16].

Though variability of soil properties is inherent in nature due to geologic and pedologic soil forming factors, intensive ongoing open-cast mining, coastal erosion and seawater ingress and landslides in the crumbling hilly areas have added to the spatial variability within and amongst the agricultural fields in the region [8]. Along with, prevalence of steep slope, high rainfall, intensive cultivation, soil erosion and loss in organic matter have been related to loss in soil quality and depleting forests [17]. Changes in soil environment has resulted in degradation of ~50% of its total geographical area causing a loss of 1.1 t ha^-1^ cereals on annual production basis [18]. The impacts of human interference and consequent decline in soil quality have been so devastating that uncultivable wastelands have almost doubled and the declining soil quality has caused severe loss in crop production and destruction of local biodiversity [5, 6].

Assessment of spatial variability of important physico-chemical soil properties and their mapping is therefore crucial for monitoring the changes in soil quality with time, precision agriculture and site effective nutrient remediation in the region. Site-specific soil test-based nutrient management with variable rates has been the most common strategy for improving soil fertility [19–21]. However spatial variability assessment through geo-statistical tools help in identification and delineation of uniform and congruous management zones for addressing the soil fertility decline through development and adoption of region-specific soil and crop management practices [22–25]. Spatial variability of soil properties can be predicted through the status of unsampled locations with the use of geo-statistical tools [26]. Interpolation and kriging technique have been used for prediction of unbiased status of studied properties of unsampled locations with minimum variance [27]. Cluster algorithm, interpolation and kriging simulation techniques have been used for delineation of uniform soil management zones. The technique reduces the sampling and analysis cost and interpolates the information of limited samples data even on unsampled locations [28, 29].

Keeping the above in view, the present study was conducted in South Goa district of the state of Goa in Indian Konkan region of Western Ghats with objectives to (i) assess the spatial variability in soil properties, *viz*., pH, SOC, EC, available N, P, K, Zn, Fe, Cu and Mn (ii) to find the correlation amongst the soil properties and (iii) to develop spatial maps of soil properties and delineate the soil management zones.

## 2. Material and methods

### 2.1. Study area

**Fig 1:**
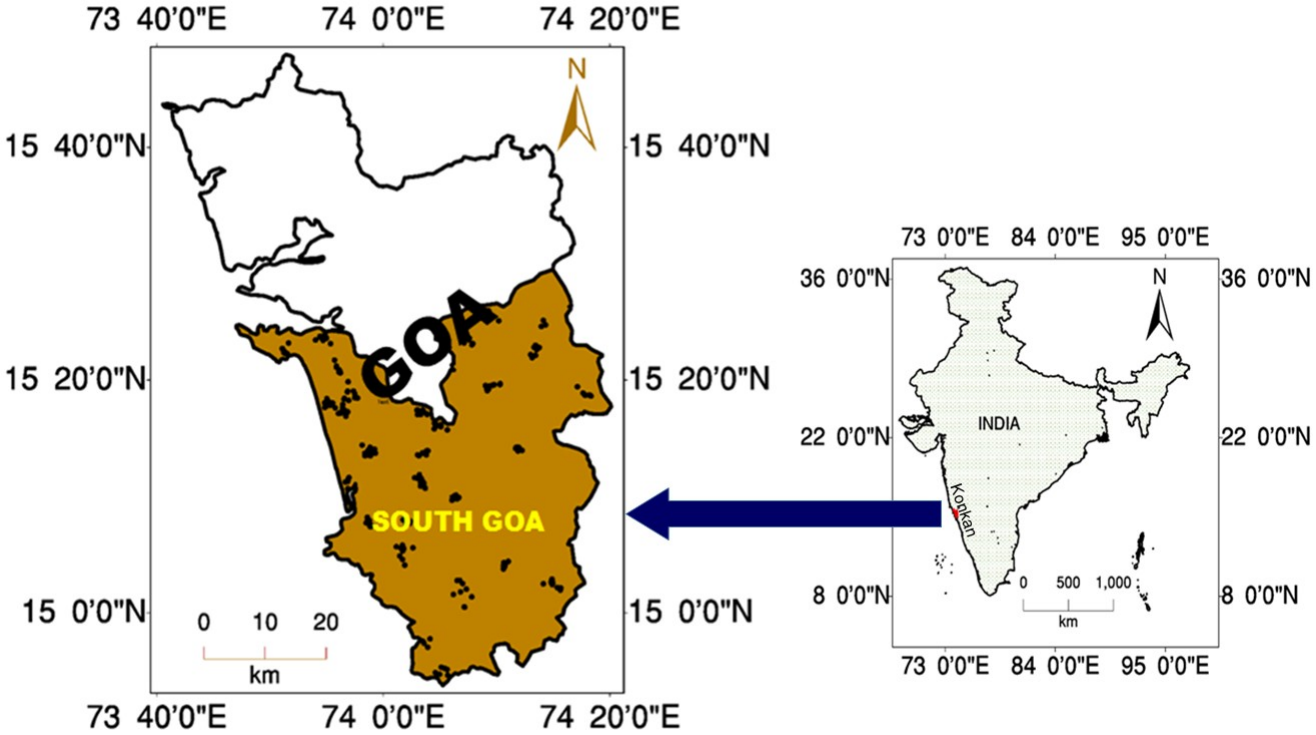
Distribution of sampling points in South Goa district of state of Goa in Konkan region of India.

The work was approved by the Director, ICAR-CCARI, Goa. The approval of the field site was also provided by the Director. The study site South Goa is located in the state of Goa in the Konkan region of Indian Western Ghats (Fig 1). It falls in the West Coast plains and Ghats region of the agro-climatic regions of India. It is one of the two administrative districts of Goa with an area of about 1966 km^2^ and bounded by North Goa district of Goa in the North, Uttara Kannada district of Karnataka in the east and Arabian Sea in the west. It is geographically lying in between 15°29’30.57" to 14°59’43.46" N latitudes and 73°47’1.24" to 74° 38’ 55.20" E longitudes and comprises three types of landforms *viz*. low lands, plateaus and mountains. Low lands are fertile, plateaus are rich in laterite stones and mountains are covered with dense forest. The major rivers are Zuari, Talpona, Sal and Galgibag.

The alluvial and red soils are classified into Inceptisol, *Ultisol*, *Entisol* and *Alfisol* soil orders and have been divided into 18 soil series [8, 30]. The textures of these soils vary from sandy loam to silty loam. These soils are developed from the parent material of granite-gneiss, quartzite/ schistose and basalt. The climate is monsoon type and hot humid situation prevails almost throughout the year. Rainfall is received by south-west monsoon during the first week of June to September and sometime extends up to mid of October. The average annual rainfall received is about 2900 mm in unimodal pattern. The warmest month is May (36°C) and the coolest month is January (19.0°C). However, December and January both are the pleasant months because of low temperature and relative humidity.

Rice, the staple food of native population, is the dominant field crop grown in plains as well as in undulated terrains on terraces. Largely grown during monsoon months as a single crop in a year, few areas with assured and adequate irrigation also go for two consecutive rice crops. The plantation crops have very good coverage in the region. Coconuts are grown on plains and low lands, however upper undulated hilly terrains are covered with cashew. The plain irrigated areas are covered mainly with vegetables while lot of area is left fallow during post monsoon period. The other major crops are groundnut, arecanut, cashew, jackfruit, sugarcane, mango, banana, pineapple, oil palm, pepper, vegetables (brinjal, okra, radish, cucumber, pumpkin, drumsticks, breadfruit, gourds, sweet potato, chilly, and onion etc.), pulses (horse-gram) and millets (ragi, maize, sorghum, pearl millet). Besides, the District also has a forest area spread across 90,491 ha.

### 2.2. Soil sampling and analysis

A total of 258 geo-referenced composite surface (0–15 cm depth) soil samples were collected following stratified random sampling procedure. The geographical coordinates, latitude and longitudes were recorded with hand-held Global Positioning System (Garmin, model e Trex Vista HCx). The collected soil samples were brought to the laboratory and air-dried at ambient temperature (25±2°C). The pebbles and debris were removed from the samples, ground and passed through 2-mm sieve. Well-processed samples were stored in polythene bags until used for analysis of soil properties. Soil pH and electrical conductivity (EC) were measured in 1:2.5 (w/v) soil water suspensions with the help of a digital pH and conductivity meter [31]. Soil organic carbon (SOC) was determined through oxidation with potassium dichromate and subsequent titration with ferrous ammonium sulphate [32]. The available nitrogen (AN) was determined by following alkaline potassium permanganate (KMnO_4_-N) method through automatic nitrogen distillation and manual titration [33]. The available phosphorus (AP) was extracted with Bray’s P-1 reagent [34] and estimated using a UV-Visible spectrophotometer. Available potassium (AK) was extracted with neutral normal ammonium acetate solution by displacement of the exchangeable cations and estimated using flame photometer [31]. Diethylene-triamine-penta-acetic acid (DTPA) extractable micronutrients, zinc (Zn), copper (Cu), iron (Fe) and manganese (Mn) were extracted with DTPA solution [35] and the concentrations of these micronutrients were determined using atomic absorption spectrophotometer.

### 2.3. Statistical analysis

The descriptive statistical analysis of studied soil properties, including the parameters of minimum, maximum, mean, kurtosis, skewness, standard deviation (SD) and coefficient of variation (CV) was performed. Relationships among the soil properties, was analyzed through Pearson’s correlation coefficient analysis. Analyses of the soil data was performed with XLstat 2018 software.

### 2.4. Geo-statistical analysis

The geo-statistical analysis was done with GIS software ARC map 10.2 developed by ESRI. The ordinary kriging (OK) with interpolation was used to decide the best suitable semivariogram models for spatial mapping of predicted data of soil properties. The normality test was performed prior to the geo-statistical analysis of the soil properties data. Moran-I auto correlation was done to find out whether data is normally distributed or not. The best fit semivariogram models were selected based on the cross validation technique. Semivariograms provide attributes like nugget, partial sill, range and log values which were used to measure the spatial variability mapping of analysed soil properties by interpolation and kriging technique [36, 37]. The semivariogram of studied soil properties were derived by using the following formula:

$$\gamma (h)=\frac{1}{2m(h)}\sum_{i=1}^{m(h)}{\left[Z(Xi+h)-Z(Xi)\right]}^{2}\ldots .$$ (1)

Where,

y(h) = Experimental semivariogram

h = Lag

m (h) = Number of sample value pairs separated by h

Z(Xi), Zxi+h) = Sample values at two points at Xi and (Xi+h) locations.

Distance between the sample pairs is rarely equal to h in irregular sampling and h is often represented by a distance interval. The comparison criteria to predict the accuracy of semivariogram model was based on mean square error (MSE) given below [38]

$$\text{MSE}=\frac{1}{\text{N}}\sum_{\text{i}=1}^{\text{N}}{\left[\text{Z}({\text{X}}_{\text{i}})-\hat{\text{Z}}(\text{X})\right]}^{2}\ldots \ldots$$ (2)

Where, Z(Xi) is the observed value at location i, $\hat{\text{Z}}(\text{X})$ is the predicted value at location i and N is the total number of samples.

### 2.5. Principal component analysis

Principal component analysis is a multivariate technique, which was used to extract factors from a set of variables. Data transformation was done by rotating the orthogonal coordinates in order to apportion variance to different components. This analysis technique works in such a way that higher magnitude of variance was loaded on the first component and subsequent lower magnitude of variance was loaded in the subsequent components. A covariance matrix, in place of correlation matrix of selected soil properties was used as input for normalized PCA analysis. The principal components (PCs) variables were included in the analysis and the PCs receiving high eigenvalues were assumed to best represent the soil properties [39]. The PCs eigenvalue equal or more than one, were considered to develop the management zones -MZs [40, 41]. The PC loading of studied soil properties have been depicted with bi-plot for understanding the variability importance of properties under various PCs.

### 2.6. Fuzzy cluster algorithm analysis

The most commonly used k-means fuzzy clustering technique was used for the development of different management zones. The fuzzy clustering technique is an extension of traditional clustering method in which a sample is forced to fall in one of the selected number of clusters by certain algorithm. This technique of clustering allows a sample with multiple attributes belonging to different clusters at the same time and lag assign membership to different groups that allows to reduce the distortion by outliers [42]. We used the three clusters as minimum and eight clusters as maximum number for practical use as management zone. Membership in each cluster was determined by an iterative process beginning with a random set of cluster means. Each observation was assigned to the closest of these means. New means were recalculated for each cluster based on the distance from the observation to cluster mean. Euclidean distance was used to calculate the distance of data points according to the result of equal variance and statistical independence. FuzMe software was used for the purpose with the setting as: maximum number of iteration = 300, the stopping criterion = 0.0001, minimum number of zones 3, maximum number of zones 8 and fuzziness component = 1.5. The fuzzy performance index (FPI) and normalized classification entropy (NCE) were used for deciding the optimum number of clusters.

$$\text{FPI}=1-\frac{\text{c}}{1\text{c}-1}[1-\frac{\sum_{\text{i}=1}^{\text{c}}\sum_{\text{k}=1}^{\text{n}}{\left(\mu \text{ik}\right)}^{2}}{\text{n}}]\ldots \ldots$$ (3)

$$\text{NCE}=\frac{\text{n}}{\text{n}-\text{c}}[\frac{\sum_{\text{k}=1}^{\text{n}}\sum_{\text{i}=1}^{\text{c}}\mu \text{ikloga}(\mu \text{ik})}{\text{n}}]\ldots \ldots$$ (4)

Where, c is the number of clusters and n is the number of observations, μik is the fuzzy membership and log_a_ is the natural logarithm.

FPI measures the degree of fuzziness created by specified number of classes. Its values may range from zero to one. The values of FPI close to zero indicate very clear clustering with limited membership sharing. However, values close to one indicate that data has no distinct classes and have large membership sharing. NCE estimate the amount of disorganization developed by specified number of classes. The minimum values of FPI and NCE optimize the number of MZs. The soil properties of the developed MZs were compared with the one way ANOVA using software XLstat 2018. The MZs were delineated with Arc GIS Map 10.2 software.

## 3. Results and discussion

### 3.1. Characteristics of soil attributes

The results of descriptive analysis of soil attributes (pH, EC, SOC, AN, AP, AK, Zn, Cu, Mn and Fe) have been presented in Table 1. Coefficient of variation, skewness and kurtosis indicate the level and degree of variation in the data. Soil pH was found in acidic range (4.92±0.61) with neutral electrical conductivity (0.08±0.37 dSm^-1^). The acidic reaction of these soils is explained to be due to the acidic parent material from which the soils were developed [2]. Spatial variation in pH has been attributed to dominance of sea water, river water, intrusion of sea water and intense tidal mixing in Zuari river flowing across South Goa [5]. Similar finding has been reported in natural forests of Savanna in Northern Ghana and in coastal saline soils of Bangladesh, where the soil pH decline has been explained to be due to removal of basic cations from the surface of soils as a result of rainfall and anthropogenic activities like mining and deforestation [43, 44]. Further, factors like accumulation of sulphate containing materials, removal of CO_2_ by photosynthesis through bicarbonate degradation and decomposition of organic materials leading to formation of carbonic acid have also led to the acidic reaction of soils [45, 46]. Soil acidity influences the chemical and biological characteristics, through direct interaction of hydrogen ions (H^+^) with microbial cells causing disruption of their cell membranes and altered enzyme production. This shifts the types of microbes leading to significant changes in mineralization rate which leads to immobilization of basic nutrients and decreases the nutrient availability to plants that equates to reduced overall microbial function towards the health and productivity of soils [47, 48]. The increased acidity has caused negative effects on the vegetation in the region through reduction in availability of several essential plant nutrient elements. pH drops from 5 to 4 create severe phosphorus deficiency, Al^3+^, Mn^2+^ and Fe^3+^ toxicity and lower concentration of available P, Mo, Ca and Mg, that indicates long term risk to the local soil environment [49].

**Table 1:** Descriptive statistics of soil properties of South Goa district of state of Goa in Konkan region of India.

Soil Properties	Unit	Minimum	Maximum	Mean ±SD	CV (%)	Skewness	Kurtosis
pH	-	3.38	7.82	4.92±0.61	12.4	1.33	4.92
EC	dSm^-1^	0.01	4.65	0.08±0.37	436	10.0	108
SOC	%	0.12	5.85	1.67±1.02	60.9	1.29	2.69
AN	kg ha^-1^	56.4	622	211.2±76.9	36.4	1.62	5.13
AP	kg ha^-1^	0.54	49.7	8.4±8.2	97.9	2.10	5.50
A K	kg ha^-1^	31.5	786	202.3±137.6	68.0	1.48	2.49
Zn	mg kg^-1^	0.01	2.96	0.22±0.30	136	5.26	36.6
Cu	mg kg^-1^	0.01	7.31	0.44±0.60	135	7.33	73.4
Fe	mg kg^-1^	1.26	38.36	7.78±5.98	76.8	1.69	3.72
Mn	mg kg^-1^	0.09	24.40	7.86±5.86	74.6	0.56	-0.54

EC, electrical conductivity; SOC, soil organic carbon; AN, AP and AK represent available nitrogen, phosphorus and potassium in soil respectively; Zn, Cu, Fe and Mn represents zinc, copper, iron and manganese.

The EC value showed highest CV, skewness and kurtosis (436%, 10.0 and 108, respectively) amongst the determined soil properties that reflected a very wide variation in the data. Wide variability in EC is attributed to the low lying saline soils near to sea. However, most of the soils were neutral in salinity. Soil pH and EC were found to be similar in soils of oil palm plantations in Western Ghats of coastal India [8, 50]. A wide variability in EC (CV = 86%) has been reported in the flood plain of river Oasis in the Mongolian Altay mountains [51].

The soils were fairly high in SOC (1.67±1.02%) and ranged from 0.12 to 5.85%. The higher SOC is ascribed to accumulation, decomposition and mineralization of aboveground biomass (forest litters and crop residues) while the lower SOC content is explained by the decreased decomposition and mineralization of the biomass due to its submergence during monsoons [50]. Low SOC contents were negatively correlated to crop growth, C input and gradual dispersion of the soil aggregates that leave organic matter unprotected and susceptible to degradation at high salinity levels [52]. Our observations are in agreement with studies conducted in an eroded area of southern China where soil erosion resulted in severe loss of SOC [53, 54]. Soil erosion decreases the above and below ground biomass causing reduction in litter input. It promotes the breakdown of soil aggregates, organic mineral complexes and exposes the deeper mineral layers to the biologically active surface layer, thereby increases the loss of SOC and elevates release of greenhouse gases [15, 55, 56]. Variability and abundance of soil C and N have exerted crucial effects on carbon and nitrogen cycles at regional and global scales [57]. It has been deduced that future carbon emissions would be dependent on large stores of organic carbon in spatially distributed anthropogenic reservoirs transported from terrestrial ecosystems [58].

Soil nutrient distributions are closely correlated to soil fertility, plant growth, carbon and nitrogen bio-geo-chemical cycles that is crucial for environmental protection in the vulnerable terrestrial ecosystems. They have great impacts on greenhouse gas emissions and global climate change [59–61]. Available N, P, K varied from very low to very high (Table 1). AN varied from 56.4 kg ha^-1^ to 622 kg ha^-1^ with an average value of 211.1±76.9 kg ha^-1^. The wide variability in AN is accounted for by the increased anthropogenic inputs of nitrogen arising from fertilizer run-off, leaching of dissolved organic nitrogen and rapid transformation rates of inorganic nitrogen on surface soils and topography. Higher nitrogen level is attributed to the fact that nitrogen can return to the surface soil through plant recycling [62]. Five year changes in source and sinks of soil nitrogen were linked with the ecological functions and greenhouse gas emissions in the coastal wetlands in a Chinese Delta [63]. Lower N Contents in the region shall affect the eco-system adversely, if the ongoing anthropogenic activities remain unchecked as it is a well-known fact that lower N and P levels cause deficiency of other elements in the soils.

AP varied from 0.5 to 49.7 kg ha^-1^ with an average value of 8.4±8.2 kg ha^-1^. Low AP levels in these soils is attributed to the acidic nature of soil. Similar finding of AP has been reported in the acid soils of Western Ghats of India [2]. Detrimental effects of soil acidity on plant growth have been related to low levels of AP in some cropped soils of India [64]. AK varied widely, ranging from 31.5 to 786 kg ha^-1^ with an average availability of 202 kg ha^-1^. The medium availability of AK might be attributed to low application of potassium fertilizers, high erosion due to undulated topography coupled with heavy monsoon rainfall [65]. Lower NPK levels lead to low plant productivity while the removal of excesses of these nutrients from the soil contribute to non-point source pollution if they enter surface water and ground water through leaching, runoff and soil erosion [66–68]. The variability is ascribed to several intrinsic (such as climate, parent material, topography and soil type) and extrinsic (land use type, farming practices, and anthropogenic activities) environmental factors [69].

The availability of cationic DTPA extractable Zn, Cu, Fe and Mn were recorded with mean values 0.22±0.30, 0.44±0.60, 7.78±5.98 and 7.86±5.86 mg kg^-1^, respectively (Table 1). The values exhibited a wide range between the below and above critical limits. Zn, Cu, Fe and Mn ranged from 0.1–2.96, 0.1–7.31, 1.26–38.36 and 0.09–2.40 mg kg^-1^, respectively. The lower levels of micronutrient contents are several folds lesser than that reported in other parts of India [70]. The variability indicated widespread deficiency of Zn, Cu and Fe with CV ranging from 74.5 to 136 (Table 1). The highest CV was recorded for Zn (138%) followed by Cu (135%, Fe (76.8%) and Mn (74.6%). Spatial prevalence of micronutrients exhibited similar trends for skewness and kurtosis. The micronutrient avalialbity and its variability is ascribed to parent material, pedogenic processes, climatic conditions and anthropogenic activities [71, 72]. The wide spread deficiency of Zn was also reported by several others in acid soils of India [2, 50]. While deficiency of micronutrients has adverse effects on growth and development of plants, higher levels of Fe and Mn in soils causes poor water quality. Ferrous ions (Fe ^2+^) get oxidized at pH greater than 3.5 to ferric ions (Fe ^3+^) and get precipitated on the surrounding substrates causing unstable microhabitats [73]. Pollution of natural environment induced by the variable distribution of these metals are indestructible and toxic to the living organisms [74]. The cumulative effect of higher and lower levels of these nutrients can be seen on the depleting vegetation in the region.

### 3.2. Relationships among the soil attributes

Significant correlation existed between soils attributes (Table 2). Soil pH exhibited significant positive correlation with EC (P = 0.05), AP (0.19 at P = 0.01), AK (0.19 at P = 0.01), Mn (0.16 at P = 0.05) and Zn (0.14 at P = 0.05) and significant negative correlation with Fe (-0.37at P = 0.01). The observations indicate that AK, DTPA Zn and DTPA Mn increased with increasing soil pH whereas Fe and Cu availability increased with decreasing pH. For each unit increase of soil pH in the range from 4–9, the solubility of Fe in soil decreases by 1000 fold as compared to 100-fold decrease for Cu, Mn and Zn [75]. Significant positive correlation between soil pH and AK is reported in organic soil of Croatia [76]. However, the negative correlation between soil pH and Fe was has been reported in alluvial floodplain soils of Bihar in India [77]. The correlation amongst soil pH, EC, SOC, AP and Fe were similar to our findings in North Goa district of Goa in India [2]. Parallel to our reports on North Goa, a negative correlation of EC existed with SOC, AN, Cu, and Mn and a positive correlation with AP, AK, Zn and Fe. However, the correlations were not significant.

**Table 2:** Correlation matrix for soil properties of South Goa district of state of Goa located in Konkan region of India.

Variables	pH	EC	OC	AN	AP	AK	Zn	Cu	Fe	Mn
pH	1	0.15^a^	0.09	0.02	0.19^b^	0.19^b^	0.14^a^	-0.04	-0.37^b^	0.16^a^
EC		1	-0.07	-0.07	0.10	0.12	0.01	-0.02	0.12	-0.05
OC			1	0.33^b^	-0.23^b^	0.20^b^	0.13^a^	0.02	-0.17^b^	0.08
AN				1	-0.19^b^	0.30^b^	-0.06	-0.05	-0.11	0.18^b^
AP					1	-0.16^a^	0.01	0.14^a^	0.06	-0.15^a^
AK						1	0.08	-0.04	-0.14^a^	0.22^b^
Zn							1	0.19^b^	0.18^b^	0.16^b^
Cu								1	0.25^b^	0.02
Fe									1	-0.22^b^
Mn										1

EC, electrical conductivity; SOC, soil organic carbon; AN, AP and AK represent available nitrogen, phosphorus and potassium in soil respectively; Zn, Cu, Fe and Mn represents zinc, copper, iron and manganese.

^a^Correlation is significant at probability level of 0.05 (P = 0.05),

^b^Correlation is significant at probability level of 0.01 (P = 0.01).

SOC, the single most important parameter that reflects the soil fertility status, exhibited significant positive correlation with AN (0.33 at P = 0.01), AK (0.20 at P = 0.01), Zn (0.13 at P = 0.05) and negative correlation with AP (-0.23 at P = 0.01) and Fe (-0.17 at P = 0.01). The concentrations of Zn, Cu, and Mn increased with increase in SOC as indicated by the positive correlation coefficient. SOC supplies soluble chelating agents and reduces oxidation and precipitation of cations, resulting in increased Zn, Cu and Mn contents [78]. Similar findings have been reported for the soils of Punjab and southern India also [79, 80]. We explained that higher levels of SOC increases the nitrogen and nutrient availability through increased microbial mineralization, improved aggregate stability, cation exchange capacity, infiltration rates, drainage and airflow [2, 11]. A significant positive relationship between soil pH and EC has also been reported in agricultural fields in western Iran while a negative relationship between EC and AN has been reported in North Goa [2, 81]. AN exhibited significant positive correlation with AK (0.30 at P = 0.01) and Mn (0.18 at P = 0.01) and negative correlation with AP (-0.19 at P = 0.01). AP had positive correlation with Cu (0.14 at P = 0.05) and negative correlation with AK (-0.16 at P = 0.01). AK had significant positive correlation with Mn (0.22 at P = 0.01) and significant negative correlation with Fe (-0.14 at P = 0.05).

Zn showed significant positive correlation with Cu (0.19 at P = 0.01), Fe (0.18 at P = 0.01) and Mn (0.16 P = 0.01). Similarly Cu had positive correlation with Fe (0.25 at P = 0.01) and Fe showed negative correlation with Mn (0.22 at P = 0.01). Significant correlation of Cu with Fe and Zn has been observed in Indian temperate climatic conditions [82]. The relationship amongst the soil attributes is attributed to the anthropogenic activities that have led to alteration in dynamics of soil pH, soil erosion, accumulation and decomposition of above and belowground biomass.

### 3.3. Geo-statistical analysis

**Fig 2:**
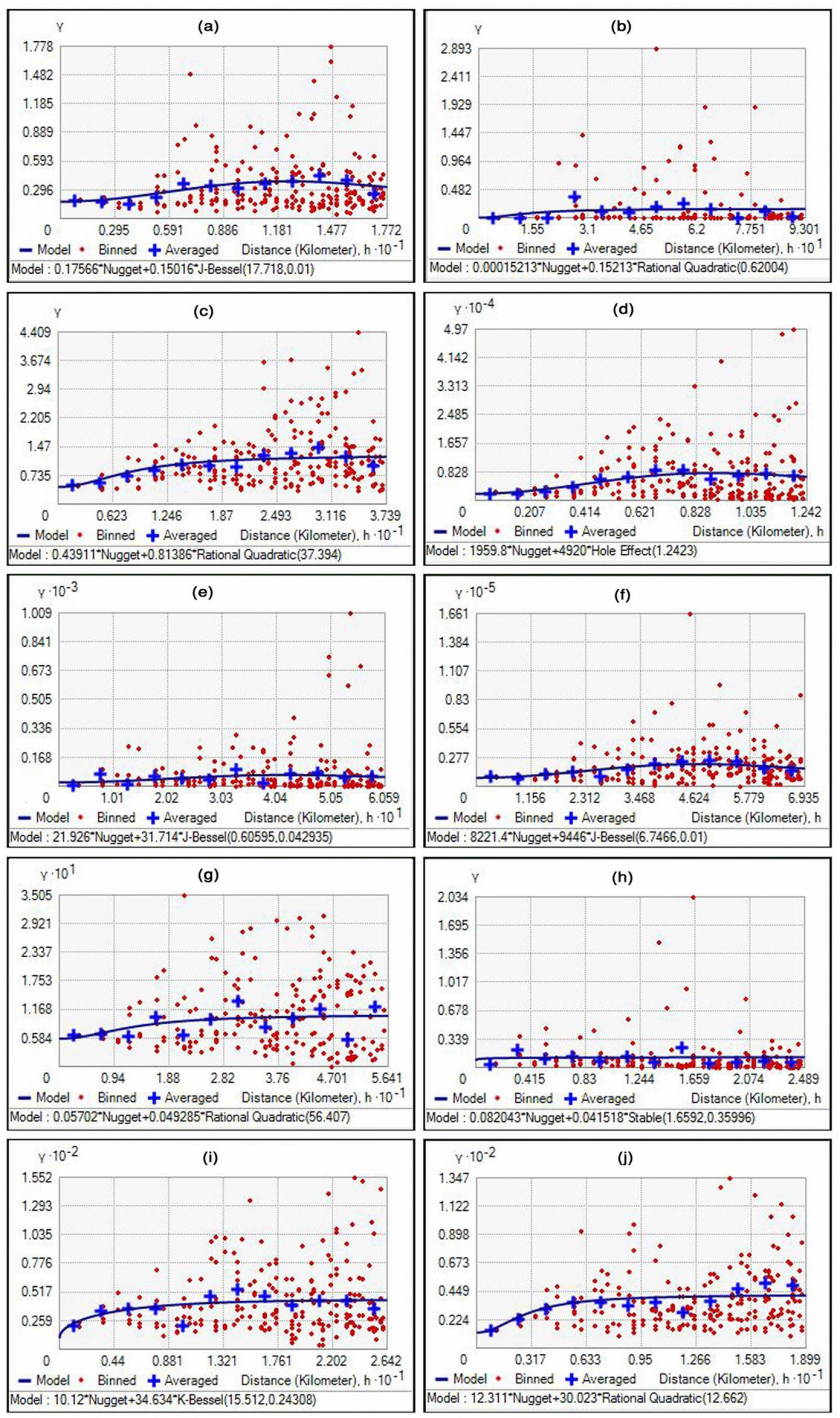
Best fit semivariogram model for soil (a) pH, (b) EC, (c) SOC, (d) AN, (e) AP, (f) AK, (g) Zn, (h) Cu, (i) Fe and (j) Mn in South Goa district in state of Goa in Konkan region of India.

The parameters of various best fit semivariogram models of soil properties are shown in Table 3. Five models, *viz*. J-Bessel, Rational Quadratic, Hole effect, Stable, K-Bessel were identified as best-fit models for the studied soil properties based on minimum MSE. The spatial distribution maps for these soil properties have been depicted in Fig 2. J-Bessel semivariogram model was found to be the best fit prediction model for soil pH, AP and AK (Fig 2a, 2e and 2f). Rational Quadratic semivariogram model was found to be best fit model for soil EC, SOC, Zn and Mn (Fig 2b, 2c, 2g and 2j). However, for AN, Cu and Fe semivariogram models *viz*., Hole effect, Stable and K- Bessel were found to be the best fit models, respectively (Fig 2d, 2h and 2i). Hole effect model for AN, spherical model for pH and K-Bessel for Fe has been reported by other researchers using similar methodology for selecting the best model for interpolation using ordinary kriging [2, 51, 77, 83]. The best fit semivariogram models for various soil properties indicate the influence of prevailing local conditions and anthropogenic activities on spatial distribution of soil properties [64].

**Table 3:** Semivariogram parameters of soil properties of South Goa district of state of Goa located in Konkan region of India.

Variables	Model	Nugget	Sill	Nugget/Sill	Spatial class	Range (km)	MSE
pH	J-Bessel	0.18	0.15	1.20	Weak	17.72	0.44
EC	Rational Quadratic	0.00	0.15	0.00	Strong	0.62	0.37
SOC	Rational Quadratic	0.44	0.81	0.54	Moderate	37.39	0.70
AN	Hole effect	1959.77	4920.00	0.40	Moderate	1.24	71.28
AP	J-Bessel	21.93	31.71	0.69	Moderate	0.61	7.24
AK	J-Bessel	8221.41	9446.96	0.87	Weak	6.75	101.52
Zn	Rational Quadratic	0.06	0.05	1.20	Weak	56.41	0.25
Cu	Stable	0.08	0.04	2.00	Weak	1.66	0.36
Fe	K-Bessel	10.12	34.63	0.29	Weak	15.51	4.72
Mn	Rational Quadratic	12.31	30.02	0.41	Moderate	12.66	4.02

EC, electrical conductivity; SOC, soil organic carbon; AN, AP and AK represent available nitrogen, phosphorus and potassium in soil respectively; Zn, Cu, Fe and Mn represents zinc, copper, iron and manganese.

Nugget value indicates the micro-variability and measurement of variance due to errors in sampling. Nugget values of best fit semivariogram models varied from 0 to 8221.4. The lowest value was recorded for EC and the highest for K. Our finding is in agreement with highest nugget value for K in alluvial soils of India that has been ascribed to the fact that the selected sampling distance could not capture the spatial dependence well [77]. Sill is theoretically equal to the variance of the sampled population at large separation distance if the data has no trend. Further, it is the semi-variance value at which the curve constantly stabilizes [84, 85]. The minimum value of sill was recorded for Zn (0.05) and highest was recorded for AK (9447). Nugget: sill ratio was classified as weak (>0.75), moderate (0.25 to 0.75) and strong (<0.25) for partial dependence [86, 87]. The data revealed weak partial dependence for pH, AK, Zn, Cu and Fe and moderate for SOC, AN and AP. However, strong spatial dependence was recorded for EC (Table 3). A higher nugget/ sill ratio indicates that the spatial variability is primarily caused by stochastic factors, such as fertilization, farming measures, cropping systems and other human activities. The lower ratio suggests that structural factors such as climate, parent material, topography, diverse vegetation, proximity to sea and anthropogenic activities play a significant role in spatial variability [88]. We too have reported moderate spatial dependence for SOC and AN in west-coast region of India [2]. A strong spatial dependence for soil AK, EC and pH and moderate for SOC and AP were reported in an organic farm of Croatia [76]. The nugget/ sill ratio values ranged from 0.00 to 0.70 with strong (for surface pH) to moderate (for SOC, AN, AP and AK) spatial dependence for the soil properties in west coast of India [8].

**Fig 3:**
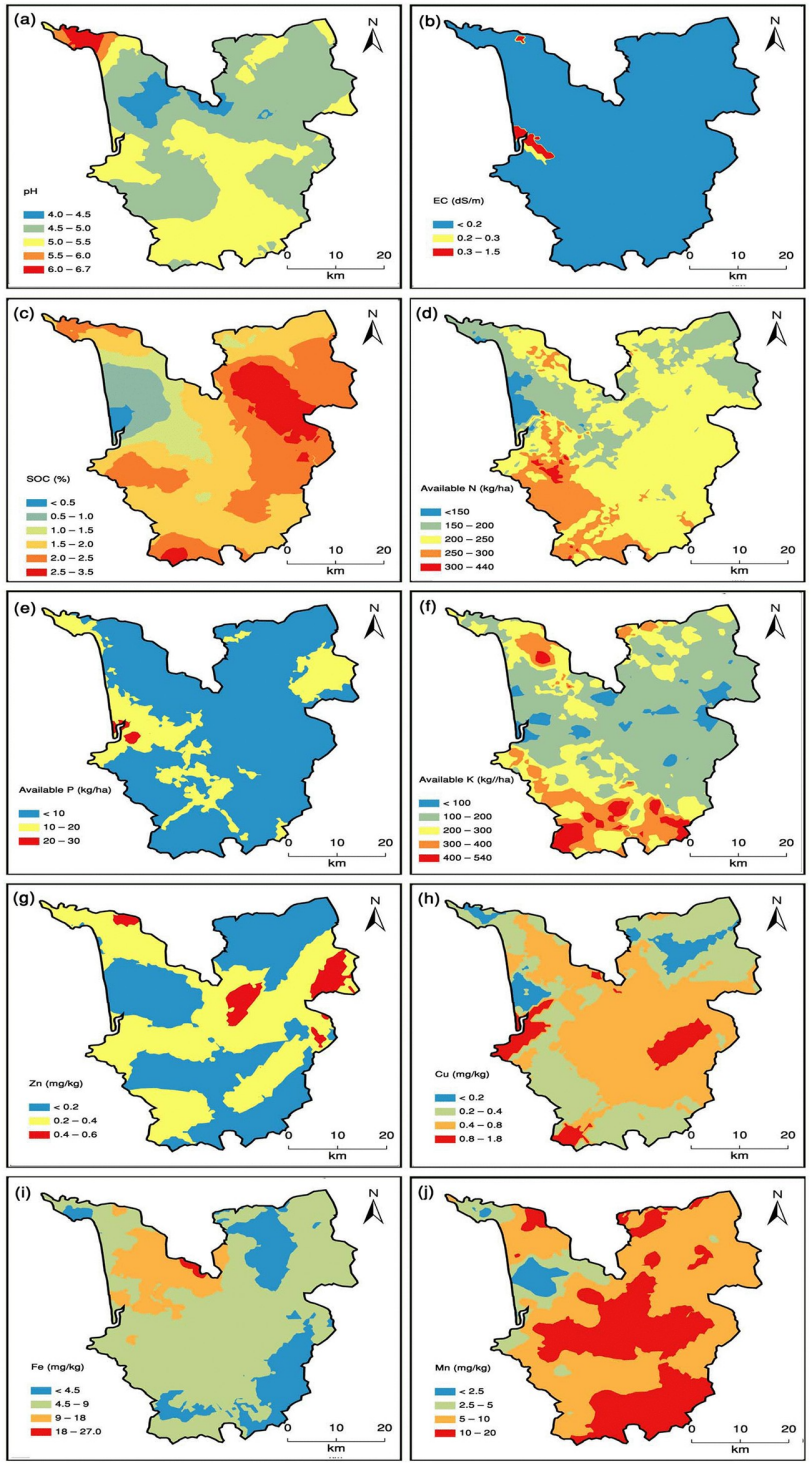
Kriged spatial distribution map of soil (a) pH, (b) EC, (c) SOC, (d) AN, (e) AP, (f) AK, (g) Zn, (h) Cu, (i) Fe, and (j) Mn in South Goa district in state of Goa in Konkan region of India.

The range of semivariogram reveals the spatial extension of autocorrelation [87]. It could either be landscape dependent or interpreted to indicate the distance across distinct soil types in the study area. The range value of best fit semivariogram models for various soil properties is presented in Table 3. The range data of semivariogram revealed variation from 0.61 km for AP to 56.41 km for Zn. Lower range values of semivariogram for soil properties like AP (0.61 km), EC (0.62 km), AN (1.24 km), Cu (0.1.66 km) and AK (6.75 km) was more spatially related than higher range values of semivariogram models. The lower range values of semivariogram models for soil properties might be attributed to inherent soil characteristics, local crop and soil management practices [88]. The higher range values for best fit semivariogram models were recorded for Mn (12.66 km), Fe (15.51 km), pH (17.72 km), SOC (37.39 km) and Zn (56.41 km). This revealed wider spatial influence of natural forces like high rainfall as well as anthropogenic activities like large-scale land excavation for ore mining in the area. The wider variation in range values of best fit semivariogram models for various soil properties have also been reported in Indian acid and coastal soils [2, 64]. The closer soil sampling distance than the range make them spatially related, whereas, those separated by distances greater than the range are not spatially related. The information generated here can be utilized further for future soil sampling strategies for spatial variability study of soil properties in the area. It is opined that for such studies, the sampling distance should be less than half of the semivariogram range [89]. Kriged spatial distribution maps of soil properties were developed to provide the present status of pH, EC, SOC, AN, AP, AK, Zn, Cu, Fe and Mn (Fig 3). Farmers, agriculture development officials, policy makers and ICT tool developers can effectively utilize the information on the developed maps for improving soil health, precision crop management, and deployment of effective input supply strategy, land use planning and designing of decision support systems. These put together would be of great help in the direction of sustainable intensification of agriculture and enriching of vegetative cover as well as bio-diversity.

### 3.4. PC analysis

The soil properties revealed significant correlations among each other. Therefore, PC analysis was performed to find out the most important soil properties which can contribute the maximum to soil quality improvement, for immediate management action to be prioritized. The PCs with eigenvalue ≥1.0 were selected for this purpose. The first four PCs showed eigenvalues ≥1.0 and a cumulative variability of 59.38% (Table 4). Therefore, these PCs were considered for further study of different soil properties. PC1 contributed 20.36% of total variability while it was dominated by loading of AK, AN, SOC and Fe. PC loading of PC2 was dominated by pH and AP. PC3 contributed 13.94% of variability while its loading was dominated by Zn, Cu and Fe. The PC4 contributed 10.79% of variability and was mainly dominated by loading of EC. This is in line with findings that reports three and four PCs from PCA by aggregating and summarizing the variability in soil properties of southern India [80] and north east Iran [90]. While determining the soil quality indices for hill-region soil of north-western Himalayas it was revealed that three PCs were responsible for 84.66% variability in soil properties [91].

**Table 4:** Principle component analysis of soil properties and loading coefficient for the first four principal components.

Principal Component		Eigenvalue			Component variability (%)			Cumulative variability (%)		
PC1		2.036			20.36			20.36		
PC2		1.428			14.28			34.64		
PC3		1.394			13.94			48.58		
PC4		1.079			10.79			59.38		
PC5		0.930			9.30			68.68		
PC6		0.851			8.51			77.18		
PC7		0.689			6.89			84.07		
PC8		0.658			6.58			90.65		
PC9		0.516			5.16			95.81		
PC10		0.419			4.19			100.0		
PC loading for each variables										
	pH	EC	SOC	AN	AP	AK	Zn	Cu	Fe	Mn
PC1	0.39	-0.07	0.58	**0.60**	-0.40	**0.61**	0.08	-0.22	**-0.56**	0.53
PC2	**0.76**	0.45	-0.22	-0.31	**0.60**	0.11	0.23	0.07	-0.27	0.13
PC3	-0.07	0.08	0.25	0.09	-0.02	0.18	**0.72**	**0.67**	**0.53**	0.20
PC4	-0.11	**0.77**	-0.02	0.21	-0.09	0.39	-0.21	-0.19	0.33	-0.27

EC, electrical conductivity; SOC, soil organic carbon; AN, AP and AK represent available nitrogen, phosphorus and potassium in soil respectively; Zn, Cu, Fe and Mn represents zinc, copper, iron and manganese.

**Fig 4:**
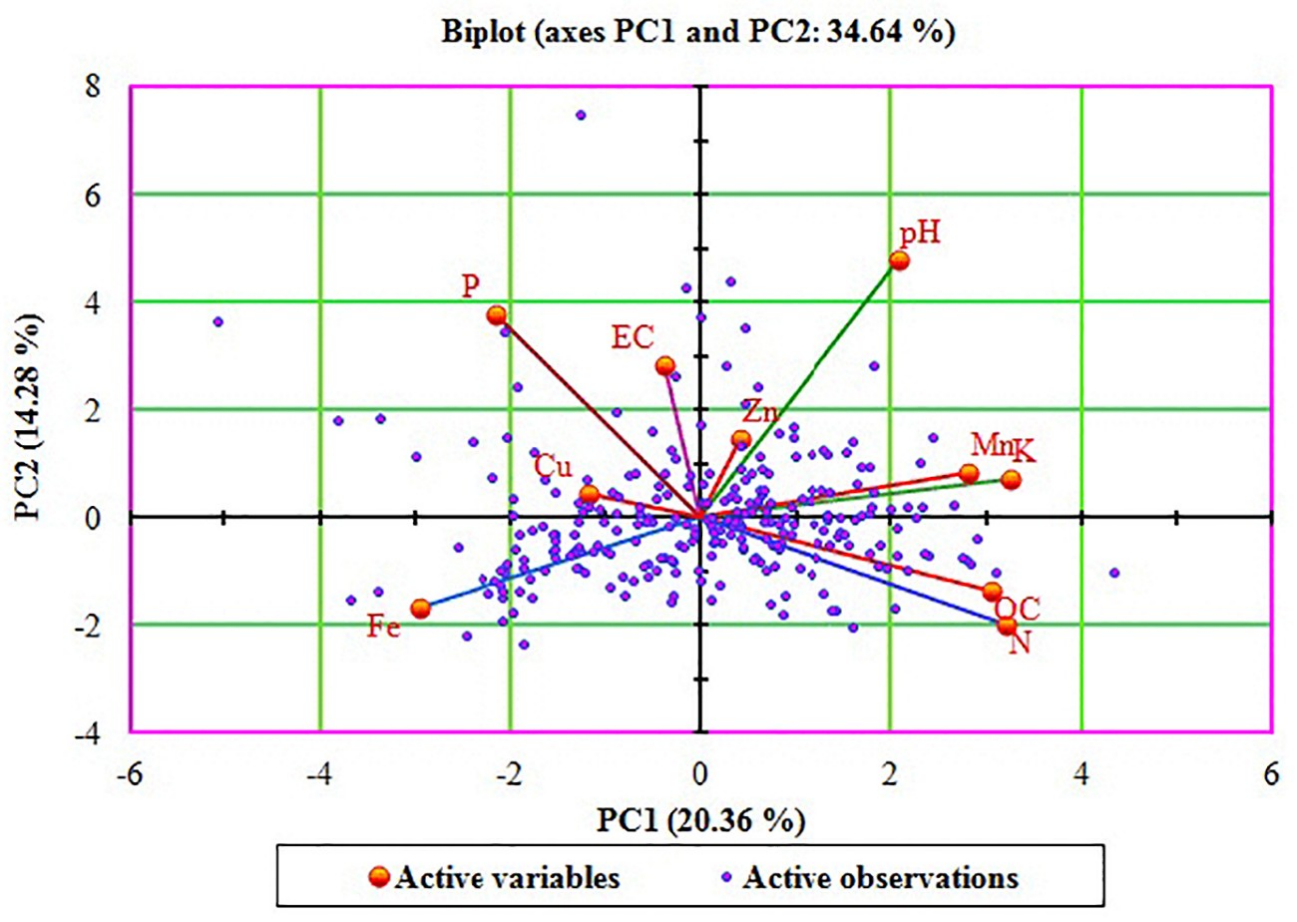
PCA biplot (PC1 vs PC 2) of soil properties in South Goa district in state of Goa in Konkan region of India.

Graphical depiction of variability contribution by PC1 and PC2 along with the loadings for different soil properties has been shown in bi-plot chart (Fig 4). Bi-plot chart elucidates the coupling of properties behaving in similar way that can be effectively bundled together for prioritization and decision making on precision nutrient management and restoration of soil fertility swiftly [2, 8]. Through expert opinion and PC loading for various soil properties, amelioration of acidic soils can be attempted. However, soil status of AP, AK and Zn content in the order may be taken as criteria to decide the prioritization of nutrient management for different crops grown in the region.

### 3.5. Determination of MZs

**Fig 5:**
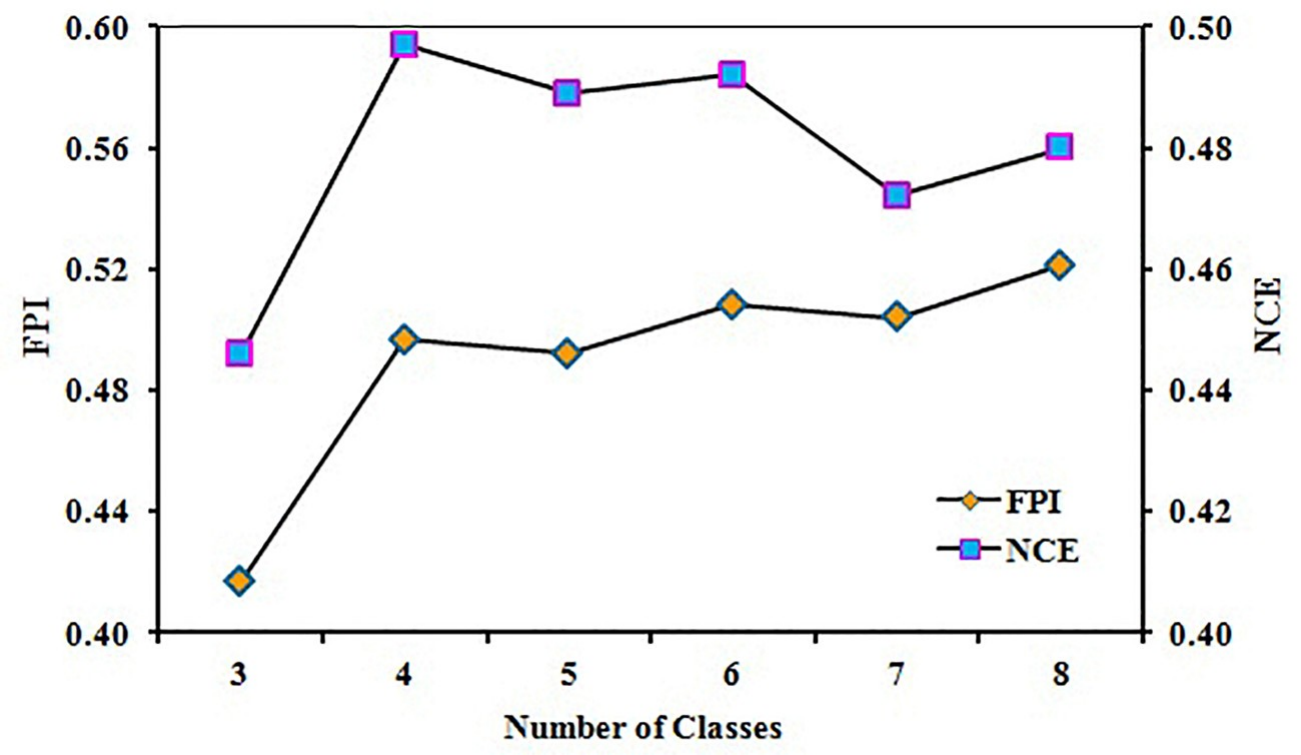
FPI and NCE for management zones optimization in South Goa district in state of Goa in Konkan region of India.

**Fig 6:**
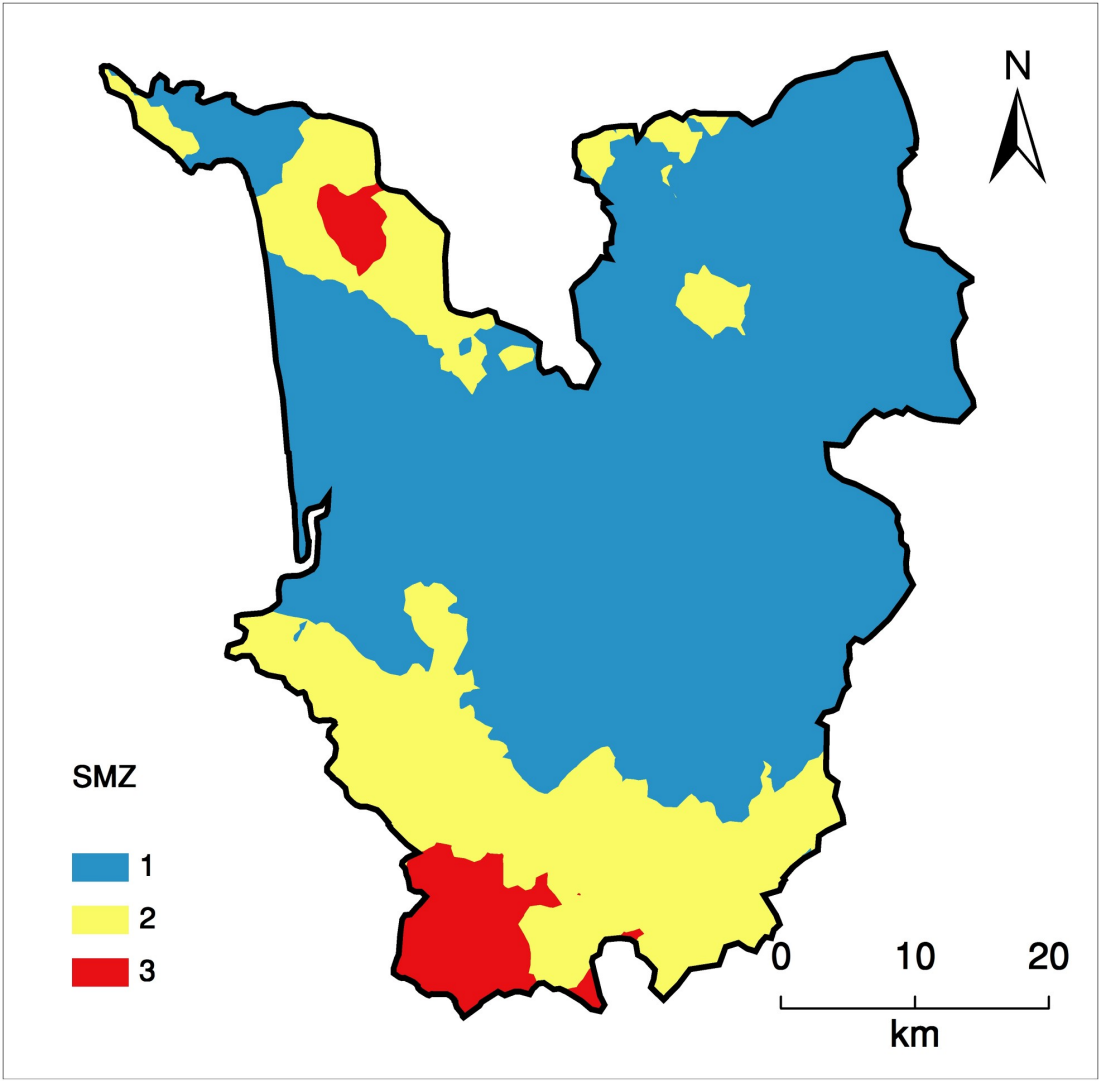
Kriged map of management zones of South Goa district in state of Goa in Konkan region of India.

Based on the k-means clustering technique three MZs were delineated (Figs 5 and 6). ANOVA of the developed MZs revealed significant differences among them for the soil properties *viz*., pH, SOC, AN, AK, Fe and Mn (Table 5). However, EC and Zn were found at par in all the MZs. Although, soil pH was found acidic in all the MZs, soils of MZ3 recorded significantly higher pH (5.1) followed by MZ2 (5.0) and MZ1 (4.8). SOC content was recorded significantly higher in MZ3 (1.93) which was at par with MZ2 (1.90). SOC content was least in MZ1. AN was recorded significantly higher in MZ3 (255.5 kg ha^-1^) which was statistically at par with MZ2 (245.3kg ha^-1^), followed by MZ1 (181.4 kg ha^-1^). AK was found significantly higher in MZ3 (518.2kg ha^-1^) followed by MZ2 (255.7kg ha^-1^) and MZ1 (110.7 kg ha^-1^). As per the classification of soil nutrient availability status in Indian conditions, low AN (<280 kg ha^-1^) and AP (<10 kg ha^-1^) was encountered in all the MZs. But AK was found in medium range (108–280 kg ha^-1^) under all MZs. Fe, Cu, and Mn were found to be above the critical limits in all the MZs but Zn was below the critical limit (0.60 mg kg^-1^) in all the MZs. Two distinctly different congruous MZs were created for North Goa district of Western Ghats [2]. The heterogeneity in soil properties in MZs is attributed to the human interference for the developmental activities and the nutrient management practices [80]. It also highlights the importance of sustaining the soil health and amelioration of soil degradation.

**Table 5:** Mean values of the soil properties in different management zones of South Goa district of state of Goa in Konkan region of India.

Management zone	No. of samples	pH	EC (dSm^-1^)	SOC (%)	AN	AP	AK	Zn	Cu	Fe	Mn
					kg ha^-1^			mg kg^-1^			
1	142	4.83^b^	0.06^a^	1.48^b^	181^b^	9.37^a^	111^c^	0.21^a^	0.41^a^	8.17^a^	6.48^b^
2	90	5.01^ab^	0.10^a^	190^a^	245^a^	7.58^ab^	256^b^	0.23^a^	0.52^a^	7.81^ab^	9.66^a^
3	26	5.10^a^	0.16^a^	1.93^a^	256^a^	5.47^b^	518^a^	0.25^a^	0.36^a^	5.56^b^	9.21^a^

EC, electrical conductivity; SOC, soil organic carbon; AN, AP and AK represent available nitrogen, phosphorus and potassium in soil respectively; Zn, Cu, Fe and Mn represents zinc, copper, iron and manganese. Values having different letters as their superscript are significantly different (P = 0.05). Comparison of values within column only.

Assessment of soil properties in different MZs brings forth the need of immediate attention for adoption of ameliorative measures in MZ1 as soils from this zone has pH <5.0, which could create unavailability of many plant nutrients and cause aluminum toxicity in soil for most of the crop plants [92]. Deficiency of AN, AP, AK and Zn in MZ1 soils calls for adoption of site-specific nutrient management in this zone. Further, delineated MZs can be used for the site-specific nutrient management to sustain the crop productivity and environmental conservation [93].

## 4. Conclusions

Our results have reflected that soil physico-chemical properties of the study area were not in good status. Mostly the soils were highly acidic but normal in salinity except in few low lying lands that were in close vicinity of sea coast. AN and AP concentrations were found to be deficient while AK showed wider variability from low to high concentrations. SOC contents were found to be fairly well but severe Zn deficiency was marked while Cu, Mn and Fe displayed sporadic deficiency. A significant spatial variability was reflected for most of the soil properties in a weak to strong spatial dependence with relatively low range values for AP, EC AN, AK and high range values for Mn, Fe, pH, SOC and Zn. Best fit geo-statistical semivariogram models *viz*., J-Bessel for pH, P and K; Rational Quadratic for EC, SOC, Zn and Mn; Hole effect for N, Stable for Cu and K-Bessel for Fe were used for developing the soil maps. Three distinct soil management zones were identified and delineated with significant variation in soil properties. The spatial distribution and mapping of soil properties will aid the farmers and decision makers in identifying the expected nutrient levels for land use planning and modify their management practices accordingly for sustainable amelioration of soil quality and environmental conservation in the region. The results can be used for making recommendations for best management practices to improve the soil health and livelihood of farmers.

## Supporting information

S1 File (XLS)

## References

[1] Singh AK. Probable agricultural biodiversity heritage sites in India: XX The Konkan region. Asian Agri-History 2014; 18 (3): 257–282.

[2] Verma RR, Manjunath BL, Singh NP, Kumar A, Asolkar T, Chavan V, et al Soil mapping and delineation of management zones in the Western Ghats of coastal India. Land Degrad Dev. 2018;29: 4313–4322. 10.1002/ldr.3183

[3] Singh SK, Ramamurthy V, Chattaraj S, Srinivas S, Hegde R. Extent and distribution of fallow lands in Goa. NBSS Publ No 173, ICAR-NBSS & LUP Regional Centre Bangalore. 2016; 264p.

[4] Nayak GN. Impact of mining on environment in Goa: A review. Environmental Geochemistry 1998; 1(2): 97–100.

[5] Gaonkar CV and Matta VM. Impact of mining on metal concentration in waters of the Zuari estuary India. Environ Monit Assess. 2019: 191: 368 10.1007/s10661-019-7506-0 31093781

[6] Reddy O, Kurothe GP, Sena RS, Harindranath DR, Niranjana CS, Naidu KV, et al Assessment of soil erosion in tropical ecosystem of Goa India using universal soil loss equation geostatistics and GIS. Indian J Soil Conser. 2016; 44 (1): 1–7.

[7] Novara A, Rühl J, Lam MT, Gristina L, La BS, Tuttolomondo T. Litter contribution to soil organic carbon in the processes of agriculture abandon. Solid Earth 2015; 6: 425–432. 10.5194/se-6-425-2015

[8] Behera SK, Suresh K, Rao BN, Mathur RK, Shukla AK, Manorama K, et al Spatial variability of some soil properties varies in oil palm (*Elaeis guineensis* Jacq.) plantations of west coastal area of India. Solid Earth. 2016: 7: 979–993. 10.5194/se-7-979-2016.

[9] Manerkar GK. Impact of mining ban on the Goan economy- A case study. Int J Comm IT & Social Sci. 2015; 2 (6): 42–53.

[10] Talule DC, Naik GR. Overall impacts of mining on the state economy of Goa: a comparative perspective of pre and post mining ban periods. Asian Journal Sci Technol 2018; 08(06): 5012–5027.

[11] Li J, Wu X, Gebremikael MT, Wu H, Cai D, Wang B, et al Response of soil organic carbon fractions microbial community composition and carbon mineralization to high-input fertilizer practices under an intensive agricultural system. PLoS ONE. 2018; 13(4): e0195144 10.1371/journal.pone.0195144 29668702 PMC5905960

[12] Rengel Z. Availability of Mn Zn and Fe in the rhizosphere. J Soil Sci Plant Nutr. 2015; 15(2): 397–409. 10.4067/S0718-95162015005000036

[13] Marshner P. Mineral Nutrition of Higher Plants 3^rd^ Edition Amsterdam Netherlands: Elsevier/Academic Press; 2012.

[14] Zhao D, Wu S, Dai E, Yin Y. Effect of climate change on soil organic carbon in inner Mongolia. Int J Climatol. 2015; 35: 337–347. 10.1002/joc.3979

[15] Zhang H, Zhuang S, Qian H, Wang F, Ji H. Spatial variability of the topsoil organic carbon in the Moso bamboo forests of southern China in association with soil properties. PLoS ONE. 2015; 10(3): e0119175 10.1371/journal.pone.0119175 25789615 PMC4366393

[16] Suh J, Kim Yi, Huiuk SM, Yosoon Choi. An Overview of GIS-Based Modeling and Assessment of Mining-Induced Hazards: Soil Water and Forest. Int J Environ Res Public Health. 2017; 14: 1463 10.3390/ijerph14121463 29186922 PMC5750882

[17] Borrelli P, Märker M, Schütt B. Modelling post-tree-harvesting soil erosion and sediment deposition potential in the Turano river basin (Italian central Apennine). Land Degrad. Dev. 2015; 26: 356–366. 10.1002/ldr.2214.

[18] Sharda VN, Dogra P. Assessment of productivity and monetary losses due to water erosion in rainfed crops across different states of India for prioritization and conservation planning. Agric Res. 2013; 2(4): 382–392. 10.1007/s40003-013-0087-1

[19] Alivelu K, Srivastava S, Subba Rao A, Singh KN, Selvakumari G, Raju NS. Comparison of modified Mitscherlich and response plateau models for calibrating soil test based nitrogen recommendations for rice on *Typic Ustropept*. Commu Soil Sci Plant Anal. 2003; 34: 2633–2643. 10.1081/CSS-120024790

[20] Mckinion JM, Jenkins JN, Akins D, Turner SB, Willers JL, Jallas E, et al Analysis of a precision agriculture approach to cotton production. Comput Electron Agric. 2001: 32: 213–228. 10.1016/S0168-1699(01)00166-1

[21] Das DC. Comparative growth analysis of *Callistephus chinensis* L. using vermicompost and chemical fertilizer. Int J Bioassays. 2013; 2(2): 398–402.

[22] Mueller TG, Hartsock NJ, Stombaugh TS, Shearer SA, Cornelius PL, Barnhise RI. Soil electrical conductivity map variability in limestone soil overlain by loess. Agron J. 2003; 95: 496–507. https://dl.sciencesocieties.org/publications/aj/abstracts/95/3/496

[23] Pereira P, Cerdà A, Úbeda X, Mataix-Solera J, Martin D, Jordán A, et al Spatial models for monitoring the spatio-temporal evolution of ashes after fire: A case study of a burnt grassland in Lithuania. Solid Earth. 2013; 4(1): 153–165. 10.5194/se-4-153-2013

[24] Xin-Zhong W, Guo-Shun L, Hong-Chao H, Zhen-Hai W, Qing-Hua L, Xu-Feng L, et al Determination of management zones for a tobacco field based on soil fertility. Comput Electron Agric. 2009; 65: 168–175. 10.1016/j.compag.2008.08.008

[25] Brevik EC, Calzolari C, Miller BA, Pereira P, Kabala C, Baumgarten A, et al Soil mapping classification and pedologic modelling: History and future directions. Geoderma. 2016; 264(PartB): 256–274. 10.1016/j.geoderma.2015.05.017.

[26] Peukert S, Bol R, Roberts W, Macleod CJA, Murray PJ, Dixon ER, et al Understanding spatial variability of soil properties: a key step in establishing field- to farm-scale agro-ecosystem experiments. Rapid Commun Mass Spectrom. 2012; 26(20): 2413–2421. 10.1002/rcm.6336 22976208

[27] Vieira SR, Millete J, Topp GC, Reynolds WD, Handbook for geostatistical analysis of variability in soil and climate data In: Alvarez VVH; Schaefer CEGR; Barros N.; Mello JWV; Costa JM. Tópicos em Ciência do Solo. Viçosa: Sociedade Brasileira de Ciência do Solo 2002 pp 1–45.

[28] Yan L, Zhou S, Feng L, Hang-Yi L. Delineation site-specific management zones using fuzzy clustering analysis in a coastal saline land. Comput Electron Agric. 2007; 56: 174–186. 10.1016/j.compag.2007.01.013

[29] Saito H, Sean A, McKenna DA, Zimmerman T, Coburn C. Geostatistical interpolation of object counts collected from multiple strip transects: Ordinary kriging versus finite domain kriging. Stoch Environ Res Ris Assess. 2005; 19: 71–85. 10.1007/s00477-004-0207-3

[30] Bhattacharyya R, Ghosh BN, Mishra PK, Mandal B, Rao CS, Sarkar D, et al Soil degradation in India: Challenges and potential solutions. Sustainability. 2015; 7: 3528–3570. 10.3390/su7043528.

[31] Jackson ML. Soil chemical analysis. Prentice Hall of India Pvt Ltd: New Delhi; 1973.

[32] Walkley AJ, Black IA. An examination of the Degtjareff method for determining soil organic matter and a proposed modification of the chromic acid titration method. Soil Sci. 1934; 37:29–38. 10.1097/00010694-193401000-00003

[33] Subbiah BV, Asija GL. A rapid procedure for the determination of available nitrogen in soils. Curr Sci. 1956; 25: 259–260.

[34] Bray RH, Kurtz LT. Determination of total organic and available forms of phosphorus in soils Soil Sci. 1945; 59: 39–45. 10.1097/00010694-194501000-00006

[35] Lindsay WL, Norvell WA. Development of a DTPA soil test for zinc iron manganese and copper. Soil Sci Soc Am J. 1978; 42: 421–428. 10.2136/sssaj1978.03615995004200030009x

[36] Goovaerts P. Geostatistics for natural resources evaluation. Oxford Univ Press New York; 1997.

[37] Tesfahunegn GB, Tamene L, Vlek PLG. Catchment-scale spatial variability of soil properties and implications on site-specific soil management in northern Ethiopia. Soil Tillage Res. 2011; 117: 124–139. 10.1016/j.catena.2011.01.013

[38] Utset A, Lopez T, Diaz M. A comparison of soil maps kriging and a combined method for spatially prediction bulk density and field capacity of Ferralsols in the Havana-Matanaz plain. Geoderma. 2000; 96: 199–213. 10.1016/S0016-7061(99)00055-5

[39] Schepers AR, Shanaham JF, Liebig MA, Schepers JS, Johnson SH, Luchiari JA. Appropriateness of management zones for characterizing spatial variability of soil properties and irrigated corn yields across years. Agron J. 2004; 96: 195–203. 10.2134/agronj2004.1950

[40] Jolliffe IT. 1986 Principal component analysis and factor analysis In Principal Component Analysis, Springer, New York: 1986; pp 115–128.

[41] Davatgar N, Neishabouri MR, Sepaskhah AR. Delineation of site specific nutrient management zones for a paddy cultivated area based on soil fertility using fuzzy clustering. Geoderma. 2012; 173–174: 111–118. 10.1016/j.geoderma.2011.12.005

[42] Brown DG. Classification and boundary vagueness in mapping presettlement forest types. 1998; 12(2): 105–129. 10.1080/136588198241914

[43] Haque MA. Variation in salinity through the soil profile in south coastal region of Bangladesh. J Bangladesh Acad Sci 2018; 42(1): 11–23.

[44] Biyogue DN. Impacts of anthropogenic activities on physical and selected chemical properties of soils in the natural forest-savanna of Northern Ghana. J Soil Sci Environ Manag. 2016; 7(5): 53–63. 10.5897/JSSEM2015.0542

[45] Rajasegar M. Physico-chemical characteristics of the Vellar estuary in relation to shrimp farming. J Environ Biol. 2003; 24: 95–101. 12974418

[46] Islam MS, Ahmed MK, Al-Mamun MH, Masunaga S. Trace metals in soil and vegetables and associated health risk assessment. Environ Monit Assess. 2014; 186: 8727–8739. 10.1007/s10661-014-4040-y 25204898

[47] Chaparro JM, Sheflin AM, Manter DK, Vivanco JM. Manipulating the soil microbiome to increase soil health and plant fertility. Biol Fertil Soils. 2012; 48: 489–499. 10.1007/s00374-012-0691-4

[48] Birgander J, Reischke S, Jones DL, Rousk J. Temperature adaptation of bacterial growth and C-14-glucose mineralisation in a laboratory study’ Soil Biol Bioch. 2013; 65: 294–303. 10.1016/j.soilbio.2013.06.006

[49] Brady NC, Weil RC. The nature and properties of soils (14th revised ed). Noida India 2013; Dorling Kindersley Pvt. Ltd.

[50] Mahajan GR, Manjunath BL, Latare AM, D’Souza R, Vishwakarma S, Singh NP. Fertility status of the unique coastal acid saline soils of Goa. J Indian Soc Soil Sci. 2015; 63(2): 232–237. 10.5958/0974-0228.2015.00031.6

[51] Jordan SG, Jannoura R, Jordan G, Buerkert A, Joergensen RG. Spatial variability of soil properties in the floodplain of a river oasis in the Mongolian Altay Mountains. Geoderma. 2018; 330(15): 99–106. 10.1016/j.geoderma.2018.05.028

[52] Egamberdieva D, Kucharova Z, Davranov K, Berg G, Makarova N, Azarova T, et al Bacteria able to control foot and root rot and to promote growth of cucumber in salinated soils. Biol Fertil Soil. 2010; 47: 197–205. 10.1007/s00374-010-0523-3

[53] Ma R, Shi J, Zhang C. Spatial and temporal variation of soil organic carbon in the North China Plain. Environ Monit Assess. 2018; 190, 357 10.1007/s10661-018-6734-z 29796954

[54] Yao X, Yu K, Wang G, Deng Y, Lai Z, Chen Y, et al Effects of soil erosion and reforestation on soil respiration organic carbon and nitrogen stocks in an eroded area of Southern China. Sci Total Environ. 2019; 683: 98–108. 10.1016/j.scitotenv.2019.05.221 31129335

[55] Ma W, Li Z, Ding K, Huang B, Nie X, Lu Y, et al Soil erosion organic carbon and nitrogen dynamics in planted forests: a case study in a hilly catchment of Hunan Province China. Soil Tillage Res. 2016; 155: 69–77. 10.1016/j.still.2015.07.007

[56] Lei L, Xiao W, Zeng L, Zhu J, Huang Z, Cheng R, et al Thinning but not understory removal increased heterotrophic respiration and total soil respiration in *Pinus massoniana* stands. Sci Total Environ. 2018; 621: 1360–1369. 10.1016/j.scitotenv.2017.10.092 29107368

[57] Mitsch WJ, Gosselink G. Wetlands 5th Edition 2015, ISBN: 978-1-118-67682-0: pp 456.

[58] Barros N, Cole JJ, Tranvik LJ, Prairie YT, Vera DB, Huszar LM, et al Carbon emission from hydroelectric reservoirs linked to reservoir age and latitude. Nat Geosc. 2011; 4: 593–596. 10.1038/ngeo1211

[59] Wang Z, Wu J, Madden M, Mao D. China’s wetlands: conservation plans and policy impacts. Ambio. 2012; 41(7): 782–786. 10.1007/s13280-012-0280-7 22457078 PMC3472011

[60] Liu ZP, Shao MA, Wang YQ. Spatial patterns of soil total nitrogen and soil total phosphorus across the entire Loess Plateau region of China. Geoderma. 2013; 197: 67–78. 10.1016/j.geoderma.2012.12.011

[61] Le Yang, Lu Fei, Zhou Xiaoping, Wang Xiaoke, Xiaonan DBS. Progress in the studies on the greenhouse gas emissions from reservoirs. Acta Ecol Sin. 2014; 34(4): 204–212. 10.1016/j.chnaes.2013.05.011

[62] Jobbágy EG, Jackson RB. The distribution of soil nutrients with depth: global patterns and the imprint of plants. Biogeochemistry. 2001; 53: 51–77. 10.1023/A:1010760720215

[63] Wang J, Wang H, Cao Y, Bai Z, Qin Q. Effects of soil and topographic factors on vegetation restoration in opencast coal mine dumps located in a loess area. Sci Rep. 2016; 16: 6 10.1038/srep22058 PMC4768095 26916152

[64] Behera SK, Shukla AK. Spatial distribution of surface soil acidity electrical conductivity soil organic carbon content and exchangeable potassium calcium and magnesium in some cropped acid soils of India. Land Degrad Dev. 2015; 26(1): 71–79. 10.1002/ldr.2306.

[65] Tripathi R, Nayak AK, Kumar DB, Shahid M, Lal B, Gautam P, et al Assessing soil spatial variability and delineating site-specific management zones for a coastal saline land in eastern India. Arch Agron Soil Sci. 2019; 65(13): 1775–1787. 10.1080/03650340.2019.1578345

[66] Wang Y, Zhang X, Huang C. Spatial variability of soil total nitrogen and soil total phosphorus under different land uses in a small watershed on the Loess Plateau China. Geoderma. 2009; 150: 141–149. 10.1016/j.geoderma.2009.01.021

[67] Yang XJ, Lin A, Li XL, Wu Y, Zhou W, Chen Z. China’s ion-adsorption rare earth resources mining consequences and preservation. Environ Dev. 2013; 8: 131–36. 10.1016/j.envdev.2013.03.006

[68] Li QQ, Luo YL, Wang CQ, Li B, Zhang X, Yuan DG, et al Spatiotemporal variations and factors affecting soil nitrogen in the purple hilly area of Southwest China during the 1980s and the 2010s. Sci Total Environ. 2016; 547: 173–181. 10.1016/j.scitotenv.2015.12.094 26780143

[69] Basso B, Liu L, Ritchie JT. A comprehensive review of the CERES-wheat-maize and rice models’ performances. Adv Agron. 2016; 136: 27–132. 10.1016/bs.agron.2015.11.004

[70] Patel KS, Chikhlekar S, Ramteke S, Sahu LB, Dahariya NS, Sharma R. Micronutrient Status in Soil of Central India. Am J Plant Sci. 2015; 6(19): 3025–3037. 10.4236/ajps.2015.619297

[71] Gadd GM. Geomycology: biogeochemical transformation of rocks minerals metals and radionuclide’s by fungi bio weathering and bioremediation. Mycol Res. 2007; 111: 3–49. 10.1016/j.mycres.2006.12.001 17307120

[72] Li J, Richter DD, Mendoza A, Heine P. Four-decade responses of soil trace elements to an aggrading old-field forest: B Mn Zn Cu and Fe. Ecology. 2008; 89(10): 2911–2923. 10.1890/07-1381.1 18959328

[73] Šimon T. Aliphatic compounds organic C and N and microbial biomass and its activity in long-term field experiment. Plant Soil Environ. 2005; 51: 276–282. 10.17221/3586-PSE

[74] Reza SK, Ray SK, Nayak DC, Singh SK. Geostatistical and multivariate analysis of heavy metal pollution of coal-mine affected agricultural soils of North-eastern India. J Indian Soc of Soil Sci. 2018; 66(1): 20–27. 10.5958/0974-0228.2018.00003.8

[75] Lindsay WL. Chemical equilibria in soils. John Wiley & Sons New York 1979.

[76] Bogunovic I, Pereira P, Brevik EC. Spatial distribution of soil chemical properties in an organic farm in Croatia. Sci Total Environ. 2017; 584–585: 535–545. 10.1016/j.scitotenv.2017.01.062 28109660

[77] Reza SK, Nayak DC, Mukhopadhyay S, Chattopadhyay T, Singh SK. Characterizing spatial variability of soil properties in alluvial soils of India using geostatistics and geographical information system. Arch Agron Soil Sci. 2017; 63: 1489–1498. 10.1080/03650340.2017.1296134

[78] White JG, Zazoski RJ. Mapping soil micronutrients. F Crop Res. 1999: 60: 11–26. 10.1016/S0378-4290(98)00130-0

[79] Verma RR, Srivastava TK, Singh KP. Fertility status of major sugarcane growing soils of Punjab India. J Indian Soc Soil Sci. 2016; 64(4): 427–431. 10.5958/0974-0228.2016.00055.4

[80] Behera SK, Mathur RK, Shukla AK, Suresh K, Prakash C. Spatial variability of soil properties and delineation of soil management zones of oil palm plantations grown in a hot and humid tropical region of southern India. Catena 2018; 165: 251–259. 10.1016/j.catena.2018.02.008.

[81] Ranjbar F, Jalali M. The combination of geostatistics and geochemical simulation for the site-specific management of soil salinity and sodicity. Comput Electron Agric. 2016; 121: 301–312. 10.1016/j.compag.2015.12.010

[82] Wani MA, Wani JA, Bhat MA, Kirmani NA, Wani ZM, Bhat SN. Mapping of soil micronutrients in Kashmir agricultural landscape using ordinary kriging and indicator approach. J Indian Soc Remote Sens. 2013; 41(2): 319–329. 10.1007/s12524-012-0242-3

[83] Pal S, Panwar P, Bhatt VK. Evaluation of spatial variability of soil properties based on geostatistical analysis: A case study in lower Shivaliks. Indian J Soil Conser. 2010; 38(3): 178–183.

[84] Mulla DJ. Modelling and mapping soil spatial and temporal variability In Henry L (Ed.) Hydropedology (pp. 637–664). Boston: Academic Press 2012 10.1016/B978-0-12-386941-8.00020-4

[85] Brito WBM, Campos MCC, Mantovanelli BC, Cunha JMD, Franciscon U, Soares MDR. Spatial variability of soil physical properties in archeological dark earths under different uses in southern Amazon. Soil Tillage Res. 2018; 182: 103–111. 10.1016/j.still.2018.05.008.

[86] Cambardella CA, Moorman TB, Parkin TB, Karlen DL, Novak JM, Turco RF, et al Field-scale variability of soil properties in central Iowa soils. Soil Sci Soc Am J. 1994; 58(5): 1501–1511. 10.2136/sssaj1994.03615995005800050033x.

[87] Oliver MA, Webster R. A tutorial guide to geostatistics: Computing and modelling variograms and kriging. Catena. 2014; 113: 56–69. 10.1016/j.catena.2013.09.006.

[88] Bhunia GS, Pravat KS, Chattopadhyay R. Assessment of spatial variability of soil properties using geostatistical approach of lateritic soil (West Bengal India). Ann of Agrar Sci. 2018; 16(4): 436–443. 10.1016/j.aasci.2018.06.003.

[89] Kerry R, Oliver M. Average variograms to guide soil sampling. J Appl Earth Obs Geoinf. 2004; 5: 307–325. 10.1016/j.jag.2004.07.005

[90] Khaledian Y, Kiani F, Ebrahimi S, Brevik EC, Aitkenhead-Peterson J. Assessment and monitoring of soil degradation during land use change using multi-variate analysis. Land Degrad Dev. 2017; 28(1): 128–141. 10.1002/ldr.2541

[91] Sofi JA, Bhat AG, Kirmai NA, Wani JA, Aabid HL, Mumtaz AG, et al Soil quality index as affected by different cropping systems in north western Himalayas. Environ Monit Assess. 2016; 188: 161 10.1007/s10661-016-5154-1 26875075

[92] Kochian LV. Cellular mechanisms of aluminium toxicity and resistance in plants. Annu Rev Plant Physiol Plant Mol Biol. 1995; 46: 237–260. 10.1146/annurev.pp.46.060195.001321

[93] Nawar S, Corstanje R, Halcro G, Mulla D, Mouazen AM. Delineation of soil management zones for variable-rate fertilization: A review. Adv Agron. 2017; 143: 17–245. 10.1016/bs.agron.2017.01.003

